# Editorial: Cognitive perception: cognition-dependent perception, perception-dependent cognition

**DOI:** 10.3389/fpsyg.2024.1371091

**Published:** 2024-05-14

**Authors:** Kristinn R. Thórisson, Pei Wang, Antonio Chella, Brendan Ruff, Helgi Páll Helgason

**Affiliations:** ^1^Department of Computer Science, Center for Analysis and Design of Intelligent Agents, Reykjavik University, Reykjavik, Iceland; ^2^Icelandic Institute for Intelligent Machines, Reykjavik, Iceland; ^3^Temple University, Philadelphia, PA, United States; ^4^Department of Engineering, University of Palermo, Palermo, Italy; ^5^Perseptio, Reykjavik, Iceland

**Keywords:** cognition, perception, general intelligence, learning, planning, prediction, artificial intelligence, unified models of mind

## Introduction

From a control-centric perspective, behavior is the result of *motor activity*, perception the result of *measurement activity*, in the broadest sense of the terms. While the latter typically requires less energy than the former, both activities depend on transduction of some sort; it is the coordination of this transduction, and its change over time based on learning and cognitive development—through the intervening cognitive activity of model creation and use—that is the focus in this Frontiers in Research Topic. We call the topic *cognitive perception* (CP).

***Cognitive perception*** focuses on coordination principles between (mid- and high-level) *perception* on the one hand and *knowledge-based control, learning* and *general cognition* on the other.

While artificial intelligence (AI) has certainly sought inspiration from the natural sciences of mind, it has yet to embrace a holistic view on how *general intelligence* is different from isolated partial solutions to particular practical problems (cf. Thórisson, 2012). In seeking a broad theory of general cognition, self-supervised learning and autonomous general intelligence (Thórisson and Minsky, 2021), the topic of cognitive perception focuses on the (bi-directional) architectural bridge between processes managing an infinite variety of information patterns coming from sensation and the creation actionable knowledge and concepts supporting achievement of low- and high-level goals, explicit and implicit.

*Perception, prediction, planning* and *learning* are categories of information management processes that determine a cognitive agent's ability to monitor the world around it, create abstracted information-based models of it, and use these models to steer immediate and future behavior. The processes in these categories are intricately intertwined. As a case in point, in the human brain the number of fibers carrying information *from* the sensory organs (‘upstream' connections) is equal to the number of fibers carrying information *to* the sensors (“downstream” connections; cf. Mesulam, 1998). Perception thus depends just as much on cognition as cognition depends on perception, even though the latter is a more commonly discussed dependency in the scientific literature on how the involved processes are coordinated.

In humans, such control and coordination processes (explicit and implicit) span up to ten orders of temporal magnitude (10 milliseconds to 80 years ≈ 9^9.4^)^1^ in three coordinated and codependent domains: (a) Immediate, situated control of an intelligent agent's body, (b) control of knowledge acquisition (world- and self-modeling), and (c) second-order control of cognitive development (developmental changes of the perception-cognition control mechanisms themselves).

A unified CP system would address the numerous open questions including representational issues, operational semantics, how the cognitive system can extract essential sparse information from a rich data stream, how it can represent perceptual data and other knowledge to provide the appropriate factorization relevant to the cognition, how it achieves attention control in complex environments, and interaction between the development of the perceptuo-cognitive apparatus and that which is thus learned — all of which rely on management mechanisms for temporally-bound resources.

While a relatively long history of research on perception and cognition in AI and cognitive science has deepened our understanding of these systems in isolation, an effective unification of the two is called for to understand the mind as a whole. Rather than being satisfied with partial understanding of only some of its parts, all of the authors in the six papers of this FrontiersIn *Special Edition: Cognitive Perception* seek a general understanding of the mind as a *whole*, presenting their arguments from various angles, on various grounds, for a unified CP agenda.

## The papers in this Research Topic

The most complete account of cognitive perception in this Research Topic is perhaps that provided by Wang et al.. Working in an epistemological context of the Non-Axiomatic Logic (NAL; Wang, 1995, 2006), grounded in the implemented Non-Axiomatic Reasoning System (NARS), the approach rests on three fundamental conjectures, namely (1) a strict experiencer-oriented view on perception and knowledge, where subjective experience is only partially transformed into objective (non-subject-dependent knowledge); and (2) active-perception view whereby the perception is entirely initiated by an experiencing agent in light of goal achievement; and (3) a unified perception-action view that assumes only a difference in quantity, not quality, between the two, that is, that perception must deal with larger amounts of data. They present results displaying many benefits over alternative methods for perceptual processing, such as deep artificial neural networks (LeCun et al., 2015).

Like Wang et al., Latapie et al. also argue that perception and cognition are not fundamentally different processes or ‘modules,' proposing instead that a key differentiator between the two is the associated attention mechanism that each calls for. This claim is quite compatible with the view that perception and cognition differ in the amount of data involved in each, since it is a fair assumption that larger amounts of data require different filtering and triggering methods, to handle false negative and positives, respectively. Both papers address questions of cumulative learning and representation. Although Latapie et al. are less strict than Wang et al. in their assumptions about underlying cognitive architecture uniformity, central to their paper is a claim that theories casting perception and cognition as separate and fundamentally different systems, e.g. that of Kahneman (2011), are themselves fundamentally misconceived and misguided.

The paper by Guillermin and Georgeon presents yet another view on the problems created by segregating perception and cognition in advancing on a holistic theory of intelligence, and discuss some of the forces in AI and robotics that are responsible for this trend over the past decades. Referring to the separation of perception, cognition, and action as *the isolated perception paradigm*, they propose to build a new unified approach on philosophical and cognitive science principles, taking a constructivist approach, that they call *Interactionist Cognitive Architecture*. Key features of their architecture include internal motivation (knowledge creation process starts from the learner's initiative, rather through events outside the learner's control), knowledge related to perception and action is stored in a unified memory, no reward functions on cognitive states are assumed (unlike in some contemporary machine learning methods), and the knowledge created is compositional – that is, it consists of a part-whole network hierarchy. They also demonstrate results from an implementation based on the approach. This *Artificial Interactionism* approach, as they call it, avoids many of the pitfalls observed in contemporary AI research, including knowledge opaqueness, lack of cumulative learning (cf. Thórisson et al., 2019a), and above all, the separation and isolation of perception from cognition.

If the concepts of ‘causality' and ‘reasoning' are rarely seen in roles of central importance in contemporary AI research, discussion about the process of ‘causal reasoning' in the AI literature are even rarer. In her developmental study, Dndar-Coecke presents work on answering to what extent a capacity for causal reasoning is related to general intelligence. Using ANOVA and factor analysis on a cohort of 138 children, the results support the hypothesis that overall cognitive ability is closely related to an ability to produce proper explanations of observed physical events that require identifying key causal relations.

Mondal takes a novel approach to CP unification in his paper, using as a point of entry the novel idea of analyzing words describing emotional concepts to expose shared operating and interacting principles of perception and cognition, the hypothesis being that through such identification their integration can be understood. The intersection of representations for linguistic knowledge and perceptual knowledge becomes in this way a source of information for a unified knowledge representation scheme related to emotion, a claim which is supported by some research in neurology (Pessoa, 2015) and constructivist theories of learning (Lindquist et al., 2015).

The sixth and final paper in this FrontiersIn *Research Topic: Cognitive Perception*, by Andonovski, is also about reasoning, more specifically about manipulable (compositional) knowledge representation of episodic information, and how this is used in reasoning for various purposes. It details how mental models of episodes – simplified information models that capture certain structural aspects of events – enable the production of simulations of (hypothetical) spatio-temporal events and reasoning over their various aspects.

## A unified theory of intelligence

In his book *Unified Theories of Cognition*, the late Allen Newell (1994) called on the fields of cognitive science and artificial intelligence to address the big challenge at the center of these fields: Understanding mind in its entirety. This was no doubt also the aim of the late AI founding father Marvin Minsky's theory Society of Mind (Minsky, 1986). It is clear from the more than 150 years of psychological research to date, however, that the field is progressing slowly toward this goal, if at all (cf. Thórisson and Minsky, 2021). In our view, building larger and more encompassing theories – even when clearly incorrect – is more promising than what both psychology and artificial intelligence are guilty of: Slicing and dicing their subject matter to (questionably) “manageable” bits. While this often makes it look like progress is being made – and admittedly this is often the case, on the bits individually that is – it is not indicative of progress toward the larger goal, as the key feature of study, *cognition*, has been chopped up so fine-grained that any and all hope of putting the partial progress together for a larger, more encompassing theory, is precluded (cf. Thórisson and Minsky, 2021).

The papers included here are all subject to certain limitations in their scope, they are concerted attempts at zooming out from the all-too-common myopic stance by addressing a larger part of the phenomenon of interest. Future research should focus on the following unifying topics:

- Compositionality and constituents of perceptual representation (cf. Zhou et al., 2022).
- The role and interplay of reasoning and statistical properties (cf. Eberding and Thórisson, 2023).
- Developmental cognitive perception (cf. Thórisson, 2022).
- Seed-programmed bootstrapping of perceptual learning (cf. Thórisson, 2020).
- Unsupervised learning of task-relevant representations (cf. Steunebrink et al., 2016).
- Attentional mechanisms in perception (cf. Helgason et al., 2012).
- Generality and autonomy in perception-based learning (cf. Thórisson et al., 2019b).
- Active perception and dynamic resource allocation (cf. Noë, 2004; Nivel and Thórisson, 2013).
- The relationship between precepts and concepts (cf. Lakoff and Johnson, 1980; Barsalou, 1999).

To speed up progress toward a more complete and comprehensive theory of mind we urge the greater scientific community in psychology, cognitive neuroscience, and artificial intelligence to include as many of these as possible in their research.

## Author contributions

KT: Methodology, Supervision, Conceptualization, Project administration, Investigation. PW: Methodology, Conceptualization, Project administration, Writing – review & editing. AC: Methodology, Conceptualization, Writing – review & editing. BR: Writing – review & editing, Methodology, Conceptualization. HH: Conceptualization, Project administration, Writing – review & editing.

## Footnotes

^1^If we assume that the smallest temporal interval that can be reliably sensed by a human is 10 msec; although this varies with the sensory modality (cf. Arieh and Marks, 2008).

## References

[B1] Arieh Y. Marks L. E. (2008). Cross-modal interaction between vision and hearing: A speed accuracy analysis. Percept. Psychophys. 70, 412–421. 10.3758/PP.70.3.412 18459251

[B2] Barsalou L. W. (1999). Perceptual symbol systems. Behav. Brain Sci. 22, 577–609. 10.1017/S0140525X99002149 11301525

[B3] Eberding L. Thórisson K. R. (2023). “Causal reasoning over probabilistic uncertainty,” in Proceedings of Artificial General Intelligence (Stockholm), 74–84.

[B4] Helgason H. P. Nivel E. Thórisson K. R. (2012). “On attention mechanisms for AGI architectures: a design proposal,” in Artificial General Intelligence (Cham: Springer), 89–98.

[B5] Kahneman D. (2011). Thinking, Fast and Slow. New York: Farrar, Straus & Giroux.

[B6] Lakoff G. Johnson M. (1980). Metaphors We Live By. Chicago: University of Chicago Press.

[B7] LeCun Y. Bengio Y. Hinton G. (2015). Deep learning. Nature 521, 436–444. 10.1038/nature14539 26017442

[B8] Lindquist K. A. MacCormack J. K. Shablack H. (2015). The role of language in emotion: predictions from psychological constructionism. Front. Psych. 6, 444. 10.3389/fpsyg.2015.00444 25926809 PMC4396134

[B9] Mesulam M.-M. (1998). From sensation to cognition. Brain 121, 1013–1052. 10.1093/brain/121.6.1013 9648540

[B10] Minsky M. (1986). Society of Mind. New York: Simon & Schuster.

[B11] Newell A. (1994). Unified Theories of Cognition. Cambridge, MA: Harvard University Press.

[B12] Nivel E. Thórisson K. R. (2013). “Towards a programming paradigm for control systems with high levels of existential autonomy,” in Proceedings of the Sixth Conference on Artificial General Intelligence, eds. K. U. Khnberger, S. Rudolph, and P. (Beijing: Wang Springer), 78–87.

[B13] Noë A. (2004). Action in Perception. Cambridge, MA: MIT Press.

[B14] Pessoa L. (2015). A network model of the emotional brain. Trends Cogn. Sci., 21, 357–371. 10.1016/j.tics.2017.03.002 28363681 PMC5534266

[B15] Steunebrink B. R. Thórisson K. R. Schmidhuber J. (2016). “Growing recursive self-improvers,” in Proceedings of Artificial General Intelligence (New York, NY: Springer-Verlag), 129–139.

[B16] Thórisson K. R. (2012). A new constructivist AI: from manual construction to self-constructive systems. Theoret. Found. Artif. General Intellig. 4, 145–171. 10.2991/978-94-91216-62-6_932175718

[B17] Thórisson K. R. (2020). “Seed-programmed autonomous general learning,” in Proceedings of Machine Learning Research, 32–70.

[B18] Thórisson K. R. (2022). “Iwssl: Introduction to this volume,” in Proceedings of Machine Learning Research (PMLR), 1–4.

[B19] Thórisson K. R. Bieger J. Li X. Wang P. (2019a). “Cumulative learning,” in Proceedings of the 12th International Conference on Artificial General Intelligence (Shenzen, China: Springer), 198–208.

[B20] Thórisson K. R. Bieger J. Li X. Wang P. (2019b). “Cumulative learning,” in Proceedings of the 12th International Conference on Artificial General Intelligence (Shenzen, China: Springer), 198–208. 10.1007/978-3-030-27005-6_20

[B21] Thórisson K. R. Minsky H. (2021). The future of AI research: ten defeasible axioms of intelligence. Proc. Mach. Learn. Res. 192, 5–21.

[B22] Wang P. (1995). Non-Axiomatic Reasoning System: Exploring the Essence of Intelligence (PhD thesis) (Bloomington, IN: Indiana University).

[B23] Wang P. (2006). Rigid Flexibility: The Logic of Intelligence. Dordrecht: Springer.

[B24] Zhou M. Duah B. Macbeth J. C. (2022). Novel primitive decompositions for real-world physical reasoning. Proc. Mach. Learn. Res. 192, 22–34.

